# Les connectivites en milieu hospitalier à Lomé: étude rétrospective de 231 cas

**DOI:** 10.11604/pamj.2018.30.176.14565

**Published:** 2018-06-26

**Authors:** Julienne Noude Teclessou, Bayaki Saka, Séfako Abla Akakpo, Houassou Matakloe, Abas Mouhari-Toure, Kousaké Kombate, Inoussa Oniankitan, Palokinam Pitche

**Affiliations:** 1 Service de Dermatologie-Vénérologie, Faculté des Sciences de Santé Lomé, Lomé, Togo; 2 Service de Dermatologie-Vénérologie, Lomé, Togo; 3 Service de Dermatologie-Vénérologie, Faculté des Sciences de Santé Kara, Kara,Togo; 4 Service de Rhumatologie, CHU Faculté de Sciences de Santé Lomé, Lomé, Togo

**Keywords:** Connectivites, Lomé, Togo, Connective tissue diseases, Lomé, Togo

## Abstract

Le but de cette étude était de documenter le profil épidémiologique, clinique, thérapeutique et évolutif des connectivites en milieu hospitalier à Lomé. Il s'agissait d'une étude rétrospective descriptive menée du 1er Janvier 1993 au 31 Décembre 2012. Elle a porté sur les dossiers des malades ayant souffert d'une connectivite dans cinq services de dermatologie et de rhumatologie des centres hospitaliers de Lomé. Au cours de la période d'étude, nous avons recensé 231 cas de connectivites dans les cinq centres d'étude, ce qui correspondait à une fréquence de 0,19% des consultations. L'âge moyen des patients était de 36,96±15 ans et la sex-ratio de 0,2. Les principales connectivites étaient la maladie lupique (50,22%); les sclérodermies (21,64%) et la polyarthrite rhumatoïde (20,35%). Sur le plan clinique, les principales manifestations cliniques des connectivites étaient les lésions de lupus discoïde (87,50%) et la photosensibilité (82,50%) chez les patients ayant un lupus érythémateux systémique; la sclérose cutanée (90,48%) chez les patients ayant une sclérodermie systémique; et une atteinte articulaire distale (100%) chez les patients ayant une polyarthrite rhumatoïde. La corticothérapie générale était le traitement de base des patients ayant un lupus érythémateux systémique (92,5%) et une polyarthrite rhumatoïde (73,47%). Les connectivites sont des affections rares à Lomé, avec une prédominance de la maladie lupique. Elles sont plus fréquentes chez la femme jeune. La corticothérapie générale reste leur traitement de base.

## Abstract (en)

This study aimed to highlight the epidemiological , clinical, therapeutic and evolutionary profile of connective tissue diseases in the hospitals setting in Lomé. We conducted a retrospective and descriptive study from January 1, 1993 to December 30, 2012. We focused on the medical records of patients with connective tissue diseases hospitalized in five Departments of Dermatology and Rheumatology at the Hospitals in Lomé. During the study period, we identified 231 cases of connective tissue diseases in the five study centers, corresponding to a frequency of 0.19% of consultations. The average age of patients was 36,96±15 years, the sex-ratio was 0,2. Major connective tissue diseases included lupus disease (50.22%), sclerodermas (21,64%) and rheumatoid polyarthritis (20.35%). Clinically, the main clinical manifestations of connective tissue diseases included discoid lupus lesions (87.50%) and photosensitivity (82.50%) in patients with systemic lupus erythematosus, skin sclerosis (90.48%) in patients with systemic scleroderma and distal joint involvement (100%) in patients with rheumatoid arthritis. Treatment in patients with systemic lupus erythematosus (92.5%) and rheumatoid arthritis (73.47%) was based on systemic corticosteroids. Connective tissue diseases are rare in people living in Lomé, with a predominance of lupus disease. They are more frequent in young women. Treatment is based on systemic corticosteroids.

## Introduction

Les connectivites ou maladies du système désignent toutes les maladies caractérisées par une atteinte inflammatoire et immunologique du tissu conjonctif. Elles ont fait l'objet d'un nombre restreint de travaux en Afrique subsaharienne, compte tenu de leur polymorphisme sémiologique qui pose des difficultés diagnostiques, de l'étroitesse du plateau technique existant, et de la sous-médicalisation des pays africains [1]. Les sclérodermies auraient une répartition homogène à travers le monde [2], avec une nette prédominance féminine. La plupart des études font état d'une inégale répartition de la polyarthrite rhumatoïde (PR) : la maladie paraît rare et peu sévère dans certains pays ouest africains [3,4]. Le lupus érythémateux systémique (LES) est réputé rare en Afrique noire alors qu'il paraît plus fréquent chez la noire américaine que chez sa compatriote de race blanche [5,6]. L'objectif de cette étude était de documenter le profil épidémiologique, clinique, thérapeutique et évolutif des connectivites en milieu hospitalier à Lomé.

## Patient et observation

Il s'est agi d'une étude rétrospective menée du 1^er^ Janvier 1993 au 31 Décembre 2012. Elle a porté sur les dossiers de malades ayant souffert d'une connectivite dans les services de dermatologie et de rhumatologie de quatre structures hospitalo-universitaires et d'un centre de dermatologie privé de la ville de Lomé. Pour chaque patient, les données collectées dans les dossiers étaient sociodémographiques, le motif de consultation, la durée d'évolution de la maladie avant la consultation, les antécédents pathologiques, les signes physiques, le(s) diagnostic(s), le(s) traitement(s) et les éventuelles complications.

**Figure 1:**
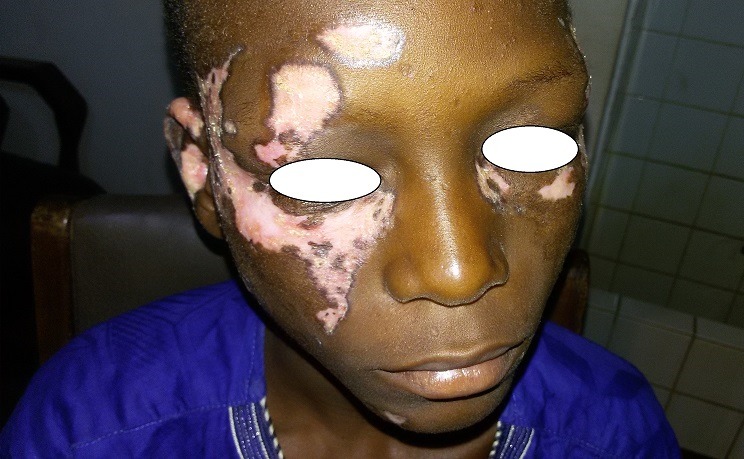
Lésions en vespertilio de la face au cours d’un lupus systémique (LES)

**Approbation éthique:** Le protocole d'étude a été soumis et a été approuvé par le comité de bioéthique du ministre de la Santé et des centres hospitaliers concernés par l'étude. Les chefs des différents services concernés par l'étude ont donnés leurs accords pour l'exploitation des dossiers. Au cours de la période d'étude, 231 (0,19%) cas de connectivites ont été recensées sur les 121021 patients reçus dans les centres concernés. L'âge moyen des patients était de 36,96±15 ans (extrêmes de 4 ans et 81 ans) et la sex-ratio de 0,2. L'âge moyen des patients de sexe féminin était significativement plus élevé que celui des patients de sexe masculin, 38,1 ans versus 31,7 ans (p = 0,01). Les principales connectivites étaient la maladie lupique (50,22 %); les sclérodermies (21,64%) et la polyarthrite rhumatoïde (20,35%). Il y avait une prédominance féminine dans toutes les connectivites (Tableau 1). Dans les formes systémiques, les signes physiques étaient dominés par les lésions de lupus discoïde et la photosensibilité (respectivement 87,5% et 82,5%) chez les patients ayant un LES (Figure 1) et la sclérose cutanée (90,48%) chez les patients ayant une SS et dans 100% chez les patients ayant une sclérodermatomyosite (Tableau 2). Les troubles pigmentaires notés chez les patients ayant une sclérodermie systémique étaient à type de poïkilodermie. Deux patients ayant une dermatomyosite avaient des d'antécédents de tumeur de la gorge et de la hanche opérée. Un patient ayant une polymyosite (PM) avait un antécédent de leucémie lymphoïde aiguë. La corticothérapie était le traitement de base chez les patients ayant un LES (92,5%), une SS (61,90%), une DM/PM (81,82%) (Tableau 2). L'évolution étaient favorable chez 47,5% des patients ayant un LES.

**Tableau 1:** Profil épidémiologiques des connectivites à Lomé (Togo)

	Nombre de cas	%	Age moyen (année)	Sex-ratio (F/H)	Durée d'évolution (%)	
					Inférieur à 6 mois	Supérieur à 6 mois
**Maladie lupique**	116	50,22				
LES	40	17,32	34,4 ±13	0,11	14,28	85,72
Lupus discoïde	70	30,30	39,2 ±13	0,15		
Lupus subaigu	6	2,60	39 ±11	0,20	30	70
**Sclérodermie**	50	21,64				
Sclérodermie localisée	29	12,55	27,9	0,45		
Sclérodermie systémique	21	9,09	31,5 ±15	0,40	34,48	65,52
**PR**	47	20,35	47 ± 12	0,18	19,05	80,95
**DM/PM**	11	4,76	29,3±12	0,83	30,95	69,05
**Sclérodermatomyosite**	5	2,16	27,2 ± 9	5/0	0	100
**ACJ**	2	0,87	13	2/0	100	0

**Tableau 2:** Aspects cliniques, thérapeutiques et évolutifs des formes systémiques

	LES	SS	Sclérodermatomyosite	DM/PM
**signes physiques N (%)**				
Polyarthralgies/Polyarthrites	39 (97,50)	-	-	6 (54,55)
Lupus discoïde	35 (87,50)		-	
Photosensibilité	33 (82,50)		-	1 (9,09)
Rash malaire	30 (75,0)		-	
Alopécie	13 (32,50)		-	
Ulcérations buccales	11 (27,50)	2 (9,52)	2 (40)	
Démence/convulsions	2 (5,0)		-	
Atteinte pulmonaire/cardiaque	2 (5,0)	2 (9,52)	-	
Phénomène de Raynaud	1 (2,50)	3 (14,29)	1 (20)	
Sclérose cutanée		19 (90,48)	5 (100)	
Trouble pigmentaire		21 (100)	4 (80)	6(54,55)
Amyotrophie			4 (80)	6 (54,55)
**Traitement N (%)**				
Corticothérapie générale	37 (92,50)	13 (61,90)	3 (60)	9 (81,82)
Antipaludéens de synthèse	17 (42,50)	2 (9,52)	-	-
Méthotrexate	2 (12,50)	1 (4,76)	1(20)	4+ (36,36)
**Evolution N (%)**				
Favorable	19 (47,50)	6 (28,57)	3(60)	5 (45,45)
Perdu de vu	10 (25,0)	15 (71,43)	2 (40)	5 (45,45)
DCD	9 (22,50)	-	-	1 (9,09)
Complications	16 (40)	-	-	

Huit cas de complications avaient été observés chez les patients sous corticothérapie. Il s'agissait d'une tuberculose péritonéale (1 cas), de septicémie (1 cas), de pneumopathie interstitielle (2 cas), d'hémorragie digestive (1 cas), de rupture d'anévrisme cérébral (1 cas), d'insuffisance rénale (1 cas) et de diabète cortico-induit (1 cas). Concernant les formes non systémiques, tous les patients ayant une PR avaient une atteinte articulaire distale avec raideur matinale de plus d'une heure (36,17%), et une déformation des articulations (27,66%). Pour le lupus érythémateux chronique (LEC), il s'agissait principalement de lésions de lupus discoïde (100%), de photosensibilité (28,57%) et d'alopécie (28,57%). Chez les patients ayant un lupus subaigu, on notait de lésions érythématosquameuses en plaques (66,67%) ou annulaires (33,33%). Les lésions de sclérose cutanée dans la sclérodermie localisée siégeaient au tronc (41,38%), aux membres (34,48%), et au visage (24,14%). Le facteur rhumatoïde était positif chez cinq des 9 patients chez qui il avait été recherché. Par rapport au traitement, 73,47% et 50% des patients ayant respectivement une PR et une arthrite chronique juvénile (ACJ) avaient reçus une corticothérapie par voie générale. Les immunosuppresseurs étaient utilisés chez 51,11% des patients ayant une PR et 50% des patients ayant un ACJ. Les dermocorticoïdes étaient le traitement de base chez les patients ayant un LEC et une sclérodermie localisée dans respectivement 42,86% et 65,51% des cas. Plus de la moitié (57,14%) des patients ayant un LEC étaient traités par des antipaludéens de synthèses L'évolution était favorable chez 88,89% des patients ayant une PR et 100% des patients ayant une ACJ. La plupart 94,49% des patients ayant un LEC et 96,55% des patients ayant une sclérodermie localisée étaient perdus de vue après leur mise sous traitement.

## Discussion

La fréquence très faible (0,19%) de connectivite de notre étude pourrait s'expliquer par la non accessibilité économique et géographique aux services de soins par les malades, mais aussi par le recours aux traitements traditionnels surtout pour des maladies chroniques [7]. L'âge moyen de nos patients ayant une connectivite en général était de 36,96±15 ans, ce qui est proche de l'âge moyen de 41,2±11,97 ans rapporté au Burkina-Faso [8]. Nous avons noté une nette prédominance féminine avec une sex-ratio de 0,2. Ouédraogo et al. [8], avait rapporté une sex-ratio de 0,16, confirmant que les connectivites sont plus l'apanage du sexe féminin. Bien que la photosensibilité soit un signe plus fréquent chez le sujet de peau blanche que chez le sujet de peau noire [9,10], elle était présente chez 82,5% des patients ayant un LES dans notre étude. Ce résultat contraste avec celui d'une étude réalisée antérieurement à Lomé [11] où la photosensibilité était présente chez 18,75% des patients, différence d'interprétation difficile. Les atteintes articulaires étaient présentes chez 97,5% et avaient inauguré le LES dans 75% des cas. L'atteinte articulaire était également rapporté dans les séries avec une proportion variant de 70,4 % à 87,75% [1,11,12]. L'atteinte rénale était retrouvée dans 27,5 % des cas dans notre série contre 37,5% dans l'étude précédente à Lomé [11] et 43,8% eu Afrique du Sud [12]. Sur le plan évolutif, les principales causes de décès dans notre étude étaient les complications infectieuses. Les infections étaient également les principales causes de décès en Afrique du Sud [12], au Gabon [13] et au Sénégal [14]. Les infections restent donc la principale cause de décès chez les patients ayant un LES sans doute lié à la corticothérapie qui constitue l'essentiel du traitement de cette maladie. Dans notre étude, nous avons recensé 21 cas de SS en 20 ans. Cette rareté des SS en milieu hospitalier a été noté en Afrique subsaharienne avec 35 cas de SS en 10 ans au Mali [15], 14 cas en cinq ans au Nigéria [16]. L'âge moyen de nos patients était de 31,5 ans et la sex-ratio de 0,4. Cet âge moyen varie de 34,4 ans à 40,3 ans dans la littérature [15,16]. Une prédominance féminine est également rapportée [16,17]. Les troubles pigmentaires étaient présents chez tous nos patients et la sclérose cutanée chez 90,48%. Les troubles pigmentaires étaient retrouvés chez 31 des 35 patients au Mali [15]; et l'atteinte cutanée était diffuse dans 57,1% des cas au Nigéria [16]. L'importance de la sclérose cutanée observée chez les africains peut laisser supposer la sévérité de cette affection en Afrique noire [18]. Concernant la PR, la raideur matinale de plus d'une heure a été signalée chez 36,17% de nos malades contre 74% dans l'étude réalisée au Sénégal [19]. Nous avons retrouvé deux cas de tumeur respectivement de la gorge et de la hanche parmi les 6 malades souffrant de la DM, ce qui rentre probablement dans le cadre des dermatoses paranéoplasiques. Cette caractéristique de la DM est similaire à celle observée dans les séries occidentales en ce qui concerne la fréquence des cancers [20,21]. Un de nos patients avait une PM associée à une leucémie aiguë lymphoïde. Hill et al. [22] avaient trouvé dans leur série une fréquence de lymphome non Hodgkinien de 3,7%, 1,7% et 8,2% respectivement dans les populations suédoise, danoise et finlandaise atteintes de PM. Ceci doit retenir l'attention du personnel de santé en particulier des rhumatologues à rechercher devant tout cas de PM, une néoplasie notamment d'origine sanguine.

## Conclusion

Cette étude nous permet de noter que les connectivites sont des affections rares à Lomé, avec une prédominance de la maladie lupique. Nos résultats confirment la fréquence des signes cutanés et articulaires comme inaugurale des connectivites. Ils rapportent également l'efficacité de la corticothérapie associée ou non aux immunosuppresseurs, mais aussi la fréquence des complications infectieuses au cours de cette corticothérapie.

## Conflits d’intérêts

Les auteurs ne déclarent aucun conflit intérêts.
